# Teachers’ and students’ perceptions of a sense of community in blended education

**DOI:** 10.1007/s10639-023-11853-y

**Published:** 2023-05-29

**Authors:** Linlin Pei, Cindy Poortman, Kim Schildkamp, Nieck Benes

**Affiliations:** University of Twente, Drienerlolaan 5, 7522 NB Enschede, The Netherlands

**Keywords:** Sense of community, Blended learning, Higher education, Social presence, Cognitive presence, Teaching presence

## Abstract

Universities have renewed interest in blended learning in preparation for post-COVID education. However, unsatisfactory social interactions hinder the quality of blended learning, despite its potential for flexible and personalized learning. In this situation, a sense of community would provide essential academic and social benefits. To develop a sense of community among students, we need to further understand students' and teachers' perceptions concerning this subject by exploring their experiences in blended learning. Therefore, we investigated this for three blended courses using a qualitative case study approach. We conducted: (1) classroom observation; (2) document analysis of course content, assignments, and assessments; and (3) individual interviews with teachers (*n* = 3) and group interviews with students (*n* = 18). The results showed the main factors that appeared to contribute to sense of community: group learning activities within courses, non-academic and extracurricular activities across courses, and the campus as a physical place integrating academic and social life after COVID. Further, we identified two obstacles: students valued group learning but struggled to manage group dynamics, and despite teachers' efforts to encourage learning autonomy, students viewed teachers as the ultimate authority in the learning process, which strained the student–teacher relationship. Additionally, this study revealed the limitations that digital tools have for promoting sense of community, as students questioned whether these tools have added value for supporting intricate and in-depth conversations. Finally, based on these findings, we provided practical recommendations for the future development of sense of community in blended learning.

## Introduction

Blended learning (BL) has been one of the fastest-growing trends in higher education for 2 decades. Prior studies have suggested BL has the potential to enhance learning effectiveness, accommodate individual needs, and improve student success (Dziuban et al., 2018; Garrison & Kanuka, 2004). Because it has the potential to combine the benefits of online education while still allowing for face-to-face contact, BL has sparked renewed interest among university teachers who are preparing for education post-COVID-19 (Lomas et al., 2021). However, some researchers have contended that BL can be a double-edged sword if its social aspect is overlooked (Moore, 2014). Poor social interactions culminate in students’ feelings of loneliness and isolation, loss of motivation, and can create barriers interfering with the quality of BL (Arslan, 2021; Hehir et al., 2021).

In this situation, a *sense of community* (SoC) is essential, representing “a feeling that members have of belonging, a feeling that members matter to one another and to the group, and a shared faith that members’ needs will be met through their commitment to be together” (McMillan & Chavis, 1986, p. 9). Evidence suggests that SoC is associated with deep learning, as well as students’ well-being (Garrison et al., 2010; Lai, 2015; Stubb et al., 2011). SoC that is conducive to learning is beneficial, but may be difficult to achieve, particularly when physical classroom time decreases in BL. Teachers must optimize their course design for BL to explicitly integrate SoC into both synchronous and asynchronous environments.

Prior research has highlighted the significance of SoC (Shackelford & Maxwell, 2012; Tayebinik & Puteh, 2015); however, limited practical guidelines are available for teachers to cultivate a SoC in BL. Moreover, students, as the most essential stakeholders, can provide recommendations to improve SoC based on their perceived experiences. To further advance BL development at universities, it is vital to consider student and teacher viewpoints. Hence, this study aims to investigate students’ and teachers’ perceptions of SoC in blended learning.

## Conceptual background

### Blended learning

In ongoing debates regarding the best definition of BL (Hrastinski, 2019), the study by Garrison and Kanuka (2004) is frequently cited, as it emphasizes the importance of thoughtful integration of classroom face-to-face (F2F) learning with online learning. A substantial amount of research has explored the capability of BL to deliver content at any time and from any place and accommodate individuals’ needs for more personalized instruction (Horn & Staker, 2017; Means et al., 2013; Osguthorpe & Graham, 2003). Other studies have further highlighted the effectiveness of BL for supporting deep learning and increasing course completion rates, retention and student satisfaction (Baepler et al., 2014; Garrison & Kanuka, 2004; Hoic-Bozic et al., 2008), making the case for further pursuing and developing BL post-COVID as well.

### Learning community

To comprehend how a sense of community develops, it is helpful to first explore the literature on learning community. Long before the advent of BL, the ability of a learning community to enhance interaction and social engagement was generally considered valuable for higher education (Zhao & Kuh, 2004). In alignment with the social constructivist theory (Dewey, 2022; Vygotsky & Cole, 1978), Berry (2017, p. 2) described a learning community as a group of “students work[ing] with peers, teachers, and staff to learn collaboratively and support each other in pursuing academic, social, and emotional goals.” Swan and Shea (2005) summarized three recurring themes of learning community in higher education: cognition in specific social situations, sharing knowledge across groups, and collaborating. Rovai (2002a) further asserted that a learning community has four components: spirit, trust, interaction, and learning. Based on these considerations, one can conclude that a successful learning community possesses the following attributes: 1) meaningful and purposeful interactions, 2) a trusting and open social climate to promote group cohesiveness, and 3) shared goals for academic success.

A learning community used to associated with a group of learners who were geographically located near each other. However, in a BL environment, a learning community’s boundary transcends both space and time. Therefore, this study adapts the definition given by Garrison and Vaughan (2011), in which a learning community in a blended context refers to a group of students sharing common academic goals and interests who are supported and guided by teachers to engage in purposeful discourse and collaborative interaction inside and outside of a classroom, through both F2F and online modes.

### Sense of community

Sustaining a learning community in BL can be a challenge due to its complexity and dynamic nature, distinguishing it from both traditional campus and fully online education. In this situation, SoC is vital due to the academic and social benefits it provides for students. SoC strengthens the learning community by cultivating meaningful interactions, collaboration, commitment to group goals, knowledge sharing, and peer support; additionally, it fosters a trusting and open social climate among students by improving their engagement, emotional connection, and wellbeing (Garrison & Arbaugh, 2007; Osterman, 2000; Rothstein & Haar, 2020; Rovai, 2002a). Rovai (2002b) explored SoC in educational settings and identified two critical variables that determine a strong SoC: *connectedness* and *learning*. *Connectedness* refers to students’ feelings of belonging and acceptance and the creation of bonding relationships, while *learning* focuses on the extent to which students realize educational goals and benefits through purposeful interaction with peers and teachers.

SoC does not occur spontaneously. For this reason, teachers must carefully design course activities that foster SoC and explicitly incorporate them into their teaching and learning practices. Studies have revealed that in order to build a strong SoC, three types of presence are beneficial in BL (learning communities), namely: students’ social and cognitive presence, and teachers’ teaching presence to establish and sustain the social and cognitive presence of students (Garrison et al., 2001; Pollard et al., 2014; Shackelford & Maxwell, 2012; Shea et al., 2005).

- The ability of the students to project themselves socially and emotionally as “real” persons in order to develop inter-personal relationships in a trustworthy environment is known as *social presence* (Anderson et al., 2019). Social presence contributes to the connectedness dimension of SoC and has three components:
  - *affective expression*, where students share personal emotions and values;
  - *open communication*, where students develop mutual awareness and recognition;
  - *group cohesion*, where students interact around common intellectual activities.
- Simply having good social relationships is not sufficient for academic success. Interactive activities in a learning community need to be structured and directed towards intellectual engagement (Garrison & Vaughan, 2011). For this, cognitive presence is necessary. *Cognitive presence* is conceptualized as the extent to which the students are able to construct and confirm meaning through sustained reflections and discourse (Garrison et al., 2001). Cognitive presence focuses on the *learning* aspect of SoC, and is a four-stage process (Garrison & Vaughan, 2011):
  - *triggering event-* defining relevant questions/tasks;
  - *exploration—*searching for valuable information;
  - *integration—*formulating solutions;
  - *resolution—*applying solutions to new situations.
- *Teaching presence* is the instruction, facilitation, and direction of students’ social and cognitive presence for the purpose of realizing personally meaningful and educationally worthwhile learning outcomes (Garrison & Vaughan, 2011). Teaching presence is usually under the leadership of teachers, and it intertwines with social and cognitive presence to create a deep and meaningful learning experience for students (Garrison & Vaughan, 2011; Xin, 2012). Teaching presence has three components (Garrison & Vaughan, 2011):
  - *instructional design and organization—*planning and designing the structure, process, interaction and evaluation aspects of a course;
  - *facilitation of productive discourse*—reviewing student comments, moving discussions forward, and checking for accurate student understanding;
  - *direct instruction*—providing intellectual and scholarly leadership to students through feedback and assessment for accurate understanding, injecting sources of information, directing discussions in useful directions, and scaffolding learner knowledge to raise it to a new level.

### Teachers’ roles, practices and challenges in cultivating a sense of community

**Fig. 1:**
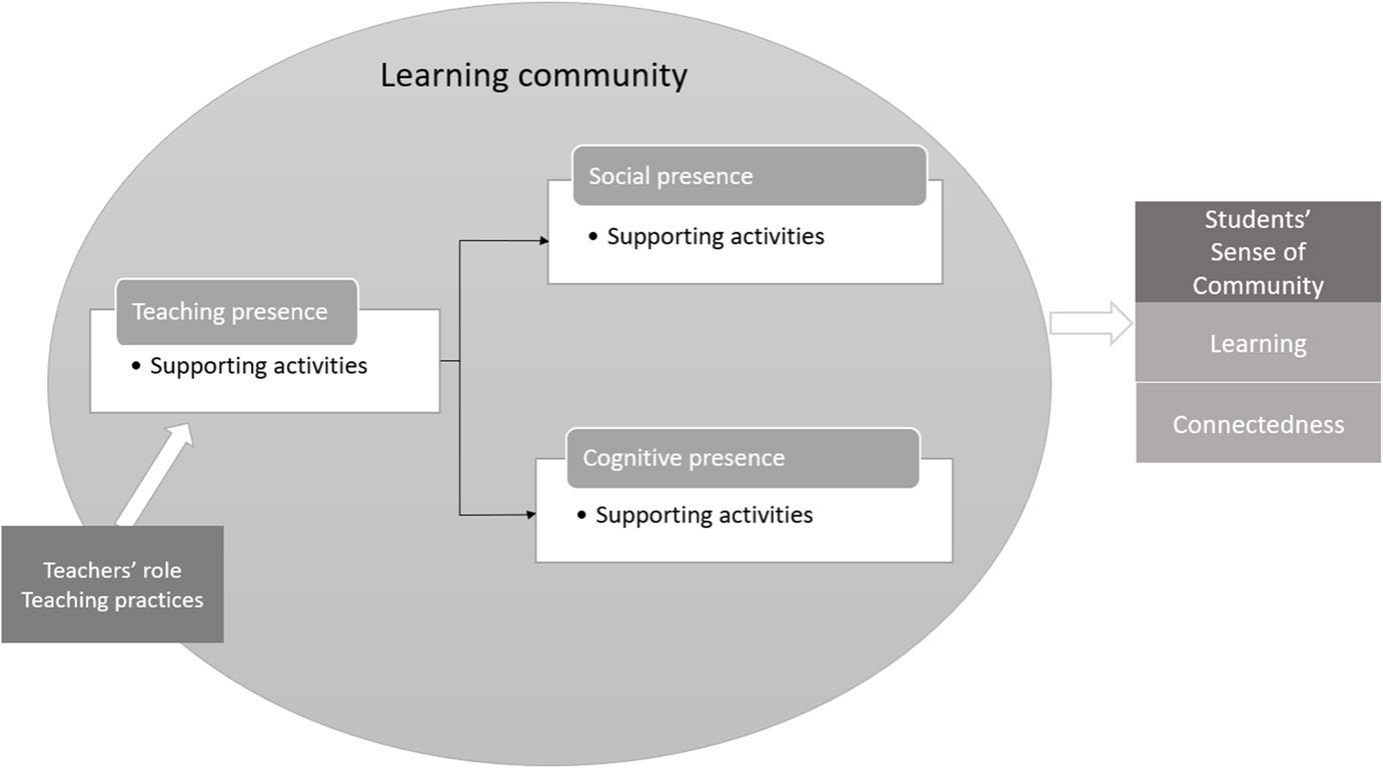
Conceptual framework

As students’ primary source of contact in a learning community, teachers play a pivotal role in helping students develop SoC. This entails adjusting teachers’ role from sage on the stage to guide on the side (King, 1993). More specifically, teachers shift from (only) direct instruction to (also) facilitating learning, as well as from knowledge dissemination to designing and supporting the learning community (Shea, 2006). From a pedagogical perspective, this in turn reshapes teaching practices in various respects, including course design, content development, deployment of learning activities and assessment methods (Norton et al., 2005; Trigwell & Prosser, 1996). Taking all of this together, prior research has demonstrated the significance of SoC in BL and the potential for social, cognitive, and teaching presence to enhance SoC. In a learning community, teachers’ roles and their teaching practices have a direct impact on teaching and learning activities in their classes, which in turn influence how students perceive a sense of community (Fig. 1).

## Research question

By incorporating the perspectives of both teachers and students, this study aims at providing a holistic picture of SoC in blended learning by addressing the question: *What are teachers’ and students’ perceptions regarding promoting SoC in a blended learning environment?* The following sub-questions are proposed:

- RQ1: What are teachers’ and students’ perceptions of SoC in BL as promoted through social presence activities?
- RQ2: What are teachers’ and students’ perceptions of SoC in BL as promoted through cognitive presence activities?
- RQ3: What are teachers’ and students’ perceptions of SoC in BL as promoted through teaching presence activities?

## Methodology

This study employed a qualitative case-study approach to gain a deeper understanding of existing views and practices of both teachers and students regarding promoting a *sense of community* in BL*.*

### Settings and participants

The study was conducted at a Dutch university that emphasizes internationalization and interdisciplinary education. Convenience sampling was employed (Etikan et al., 2016, pp. 1–4): three teachers intending to improve their BL courses that started in 2022, were invited to join this study. To ensure maximum sampling variation, teachers from distinct disciplines were chosen, and the courses varied in terms of class size, student backgrounds, and teacher prior experience in BL development (Table 1).

**Table 1:** BL course profiles

	Course A	Course B	Course C
Discipline	Social science	Technology	Engineering
Includes international students	Yes	Yes	Yes
Students from different programs	Yes	No	Yes
Teacher’s experience with BL	1 year	3 years	4 years
Number of students in course	76	51	90
Number of students interviewed	5	7	6

### Data collection

The university’s ethics committee approved this study prior to data collection. Each participant signed a consent form. To provide research triangulation, we utilized multiple methods in data collection, including non-participant observation, document analysis and interviews (Yin, 2009).

#### Non-participant observation

First, non-participant observation was conducted by gathering first-hand and unbiased “live” data in situ, rather than using self-report data only (Wellington, 2015, p. 247), to identify activities related to the three types of presence that could be fostering students’ SoC in classrooms. Nine classroom observation sessions were conducted per course, at the beginning, middle, and end, to examine SoC at various stages. An observation protocol was used (Appendix 7) that gave examples of activities that were associated with the aspects or elements of social, cognitive and teaching presence (Table 2; Garrison et al., 2006).

**Table 2:** Categories of social, cognitive, and teaching presence

Type of presence	Categories
Social Presence	Affective expression
	Open communication
	Group cohesion
Cognitive Presence	Triggering event
	Exploration
	Integration
	Resolution
Teaching Presence	Instructional design and organization
	Facilitating discourse
	Direct instruction

The semi-structured protocol consisting of two sections: capturing activities related to the three presences and elements that could impact SoC. The researcher took field notes while reflecting on the teachers’ practices that could support students’ SoC.

#### Document review

Second, course data on the learning management system (LMS) was collected, and a checklist (Appendix 8) was used to analyze the course content, assignments, and assessments to identify specific course elements influencing students’ SoC. This LMS document review was necessary because classroom observation alone would not capture the crucial component of teaching presence — instructional design and organization.

#### Interviews

The observation and document review revealed preliminary findings regarding the SoC-promoting activities that were actually implemented; interviews were then conducted to elicit more in-depth explanations from participants regarding how and why the observed activities affected SoC (Cohen et al., 2002). The interviews were semi-structured to maintain the direction and focus of our research framework. Additionally, open-ended questions allowed the participants to freely express their thoughts (Bogdan & Biklen, 1997), to explore unanticipated findings (Cohen et al., 2002). We tested the interview protocol (Appendix 9) first with a teacher to improve the clarity of the questions and increase the reliability of the data gathered. At the end of each course, we conducted one-on-one interviews with teachers and a group interview with students. Each interview lasted approximately one hour. During the teacher interviews, additional discussion took place to elicit the teachers’ perspectives on their roles, teaching practices, obstacles, and wishes regarding promoting SoC in BL. All students were approached and five to seven students per course volunteered to be interviewed in a group to determine what activities they preferred for enhancing SoC. The students interviews were also meant to discover the factors that would contribute to SoC, but were not implemented by the teachers. All interviews were recorded and transcribed verbatim for content analysis.

### Data analysis

We used content analysis of the observation, the document review notes and the interview transcripts to investigate teachers’ and students’ perspectives on SoC (Miles & Huberman, 1994). First, deductive analysis was carried out using pre-defined codes associated with the three types presence (Table 2). Second, inductive content analysis was used to generate new emergent codes by scanning collected data for new discoveries (see Appendix 10 for code book). For instance, the “group dynamic” code reflected obstacles that students have when working in groups; “different views” was related to the issue that students and teachers had varied expectations; and “student wishes” referred to students’ suggestions for improving SoC in the future. To assure coding reliability, a researcher who was not affiliated with this study coded about 10% of the total data. The inter-rater agreement was calculated as 84%.

## Results

To ensure the uniqueness of each case, within-case analysis was first undertaken to determine the stand-alone findings for each course (Appendix 11). We then conducted across-case analysis to shape commonalities and differences among all three courses (Stake, 1995), which are presented in the following section. The results were derived from classroom observations, LMS document reviews, and interviews.

### Teachers’ and students’ perceptions of SoC in BL as fostered by teaching presence activities

#### Instructional design and organization

According to the observation data, LMS information, and interviews, all three teachers used a flipped classroom strategy to support the learning aspect of SoC in designing their blended courses. They hoped students would engage in self-study by reading materials and watching videos prior to completing assignments. Teachers organized the F2F sessions in various ways, including Q&A sessions, supervised tutorials and group projects.

All of the interviewed students expressed appreciation for the flipped classroom strategy: the accessibility of online content allowed for flexible and personalized learning experiences. However, students also expressed an explicit need for in-person (F2F) interaction with their teachers to receive additional assistance with their difficulties:

*I prefer having mini-lectures and the Pencast online … if I want to write down the whole derivation, I always pause a lot and try to understand every step and .. I can go at my own speed. But tutorials in general, I don’t think you could do that online.*

LMS showed that teacher B used a learning analytics tool to gain deeper understanding of students’ learning patterns. During the interview, she stated that this was particularly essential for blended learning, in which more learning occurs outside of the classroom. Learning analytics gave insights into and visualizations of how students interacted with course content.

#### Facilitating discourse

The observations showed that all teachers encouraged students to ask questions: *“There are no wrong answers or stupid questions”*. Two teachers used the discussion board (DB) to foster student-to-student interaction. However, the students interviewed did not acknowledge the potential of the DB to promote SoC. Some mistakenly thought that only the teacher could respond to the questions and that students were not supposed to post their ideas. LMS data showed that engagement via the DB primarily occurred between individual students and the teacher. The DB was mainly used by the teachers to answer student questions rather than to foster student discourse.

During the interviews, teachers B and C said that they should take the lead in promoting SoC by organizing intentional discussions. Teacher C was observed to re-arrange the chairs to facilitate discussion, so that students sat in small circles: “*I seated, I don’t stay standing up…I’m not delivering. I’m discussing with them.”* During F2F classes of course B and C, students were observed to discuss, interact, debate and solve problems together, with the assistance and guidance of the teachers.

When the course design did not explicitly require students to engage in discussion and collaboration, students expressed a need for this type of activity. They stated during the interview, *“It would have been helpful if the teacher had… proposed for the students to work together on teams”.* Notably, students did not just wait for their teacher’s initiatives. Instead, they voluntarily formed an informal group outside of class to exchange ideas and discuss challenging topics, as they stated in the interviews.

#### Direct instruction

For all three courses, traditional classroom presentations were largely replaced by pre-recorded mini-lectures. This freed up classroom time for teachers to provide a recap of the self-study, use digital polls to identify misconceptions, give feedback on assignments, and clarify confusion among students. To establish their personal presence outside of class, the teachers provided students with email and DB options. However, the students interviewed still preferred to discuss challenging questions with their teachers during the F2F classroom sessions.

During the interviews, all three teachers considered themselves as not so much traditional lecturers. They preferred to be “*a facilitator by bringing specific topics for group discussion… observe, reflect, model, and debrief the students”.* They considered learning autonomy as important and chose to allow students to tackle problems independently first.

Teacher A was observed to use the Q&A sessions to address individual questions. She mentioned during the interview that “*I wanted students (to) think about (a question) and I then gives a hint in the right direction*.” The interviewed students were pleased with how she explained subjects and offered instruction during the Q&A sessions.

Teacher B used the classroom time for supervised tutorial sessions. The majority of the students were observed actively participating in the discussion and making efforts to assist one another. They also tried to cheer each other on by saying*: “Believe in yourself” and “Take it easy, you can do this”.* It is worth mentioning that the teacher did not make the tutoring sessions compulsory. The majority of the students, however, were present during the observed sessions. Students mentioned that these sessions fostered social relationships and enhanced their learning in the interview.

We observed in course C that each project group was asked to create rubrics and assess the work of the other groups. In the interview, the teacher hoped that “*students themselves work together to understand the assessment rubric and can propose changes they consider necessary to make it clearer.”* The students interviewed, on the other hand, had divergent viewpoints. It seemed difficult to them, and they did not believe they should be in charge of the assessment rubrics.

Despite acknowledging the importance of group interactions to promote SoC, students from all three courses expressed difficulties and dissatisfaction during the interviews. Some of them felt they received little confirmation from their teacher: they felt like *“didn’t get a very straightforward reply”* and they *“didn’t know how to continue”.* Others noticed group activities were occasionally “*chaotic”,* and they wished to have more clarification and direction from the teacher. It must be noted that students repeatedly brought up this point during the interviews.

### Teachers’ and students’ perceptions of SoC in BL as fostered by social presence activities

#### Affective expression

All three teachers were observed to greet students during the first lecture and they also posted welcome messages on the LMS. Two used funny videos and photos to introduce themselves, shared personal things like hobbies. According to the students interviewed, these initiatives helped them establish rapport with the teachers. They felt they learned more about their teacher as a person, allowing them to be more open with the teacher about their difficulties. They thought that a “*grea*t” teacher has a positive impact on their SoC. They brought up a few examples:

- A female student mentioned how “*cool*” it was to see her teacher in this technical study. She regarded her as a successful example of a woman in a technical field.
- Students named a teacher from their program and stated that they would choose his course due to his passionate teaching style, which motivated them to learn.
- Students used an example of a teacher making a mistake and quickly admitting it. Students perceived him as a real person, which helped to bridge the social divide between them and the teacher.

We noticed a natural bond among the students who were in the same academic program (those in course B). Those students mentioned in the interview that their program organized an orientation week for them to become acquainted with one another and their teachers. Teacher B joined in the orientation event voluntarily. She believed it was an excellent way to foster student–teacher rapport through icebreaker activities and said in her interview that she wished that her colleagues would participate more in this type of activity.

In contrast, courses A and C consisted of students from different faculties who had never met before. Just a few classroom activities were observed to target students’ getting acquainted. When students were required to form their own groups during the first lecture, they took a long time. It was also challenging for them to immediately begin working on a task, as some students worked alone with little interaction with others. During the interviews, the students expressed a need for more time and assistance in becoming acquainted with each other at the beginning of the course. They suggested adding a social introduction to the course, as “*that would help with creating some sort of community”*.

From a social standpoint, students acknowledged the importance of campus life in the interviews, because it fostered a sense of belonging and promoted more effective interpersonal communication: *“It’s part of the experience. It’s part of the feeling (of being on campus). If I’m staying at home, I’m lost. I feel isolated, isolated!” Students also believed that the assignments that required extensive peer interaction were a driving force behind their motivation to be on campus, thus further increasing their SoC: “We saw each other once or twice a week. And then we also had the meetings … to work on assignments… the community feeling was quite a lot”.*

#### Open communication

All three teachers were observed to urge students to openly express their ideas: *“Making mistakes is part of learning”, “Everyone is welcome in this classroom”.* Two teachers also used digital polling allowing students to ask and answer questions anonymously. During the interviews, they reported this would stimulate the more reserved students to respond to questions. However, most of the interviewed students were skeptical that digital polling would help them to be more open.

Students from all three courses reported in the interviews that they used WhatsApp groups (without teachers) to interact with one another outside of class. They were enthusiastic about its ability to promote SoC, because it allowed them to assist each other with academic as well as non-academic questions. Students expressed appreciation for their program’s support in promoting SoC, as the program established a WhatsApp group made up of students from different years, leading to the extension of SoC beyond a single class.

#### Group cohesion

According to the interviews, all teachers believed that SoC is important because it allowed students to work together “*aiming at the same goal.’’* Students agreed with this, stating that a strong sense of group cohesion is associated with group learning activities: “*You really work together and that you really have a sense of community there…if you don’t understand things, you ask a lot of people. So in a way where we’re all kind of connected”.*

When asked for specific examples, students named supervised tutorials, diagnostic tests by preparing exams together and group projects as the most significant contributors to SoC. Through these group activities, they assisted and motivated one another:

*We used to write some nice comments (to each other). I was checking the work of one girl and she was checking my work. I used to draw smiles on her homework and she wrote back: we are almost there!*

Moreover, we learned in the interviews that in course C, the interactions among students took place not only within a group, but also across groups. The students who had the same project role (e.g., project manager) from multiple groups met frequently to discuss their challenges and seek help from each other. They perceived that this type of cross-group collaboration played a big role in establishing SoC. They commented that everyone cooperated and there was less competition, but more support and understanding. Some of the students even volunteered to meet “*in the city center once, with a group just to have a few drinks. It was really nice*.”

Despite students’ enthusiasm for group cohesion, in the interviews, students from all three courses expressed struggles with managing group dynamics:

- Students had difficulty scheduling a common time outside of class for group work and not everyone was able to stick to strict deadlines.
- Some students indicated that group discussion was often *“led by a very small set of students.*”
- Some recognized that not everyone was devoted to the team’s efforts: *“if everyone is motivated, everything is fine. Someone is not motivated, that’s a challenge*.”
- Some perceived cultural differences as a challenge in communication. Some students' "*quiet*" behavior was interpreted as a lack of participation.

### Teachers’ and students’ perceptions of SoC in BL as fostered by cognitive presence activities

The data from LMS showed that the students from all three courses were given assignments to complete (*triggering event*), after which they would explore and gather relevant information from the course material (*exploration*). They then began to formulate solutions and construct their understanding by completing the assignments (*integration*), and in the final phase they would create, apply, and evaluate their solutions in new situations (*resolution*).

#### Triggering Event

According to LMS data, assignments were the primary means of triggering events to “create a sense of puzzlement” for the students (Garrison et al., 2006) in all three courses. Course A consisted solely of individual assignments, whereas courses B and C required group assignments and assessments. All three courses included real-world challenges or problem-solving tasks based on their own experiences in order to pique their interest and curiosity. For instance, in course A students were asked to formulate research questions from their own perspectives: *“Select a topic about a social problem which you care about. …Write down … about your problem”.*

*Additionally, the use of digital polls* was observed in two courses, providing teachers with insight into the students’ comprehension. During the interview students commented that digital poll helped them “*reflect on the things*” they had learned.

#### Exploration

The LMS data showed that all teachers supplied self-study content (e.g., videos, reading materials and quizzes) to help students research relevant information and explore possible solutions to the problems presented in their tasks. All three courses made extensive use of video. For example, teacher B offered Pencast videos that demonstrated how to solve equations. She recalled during the interview that if she wrote a derivation on a blackboard, students did not always follow her and would instead begin chatting, so she had to devote time to class management. She anticipated that students would be more motivated and more responsible for their own learning by studying the Pencast on their own. The possibility of watching pre-recorded videos was valued by every student interviewed. They thought their individual learning process was enhanced by *“streamlining the input and the flow of information proceed at my own pace”.*

All three teachers were observed to explore the potential of using various technologies in their courses. In the interviews, they mentioned several ICT tools (e.g., digital conference, Google Doc, and scrum board) as ways to promote student collaboration. Nevertheless, the students interviewed did not find these tools to be beneficial for collaborative activities. They preferred to keep the use of ICT tools as simple as possible, because otherwise they felt distracted and spent more time and effort adjusting to the tools than on learning. An in-person meeting was the preferred method of interaction when they needed to work together.

#### Integration and resolution

According to the observation and LMS data, integration and resolution activities frequently took place concurrently in three courses. The teachers designed assignments that progressed from simple tasks such as recalling knowledge to more difficult tasks such as integrating, evaluating and creating new solutions.

According to the students from course A who were interviewed, Q&A sessions helped them the most in their cognitive development. The classroom observation confirmed that in-depth conversations at a higher level often occurred during the Q&A sessions.

The observation and LMS data showed that course B included many collaborative problem-solving activities in group assignments and assessments. Students first completed a diagnostic test individually; they then formed groups to grade each other’s tests and provide feedback. In course C, students were observed to work in project groups to produce final products together. Teacher C stated in the interview,

*There is no escape from that. (Students) have to collaborate, they have to rely on each other because they have pieces of the puzzle that they need to create. And (effort to solve) the full puzzle is fully rewarded. So it doesn’t make sense that you have just your own piece.*

All three teachers stated in the interviews that they were satisfied with students’ performance. They felt that students were more engaged in classroom learning activities and completed given tasks at a higher level than during the COVID period.

### Teachers’ and students’ general perceptions of SoC

In the interview data, we saw that teachers in this study believed that SoC is essential for BL, because “(*students) can rely on each other… they can collaborate.*” Similar to their teachers, all students reported valuing SoC, because it provided them with the motivation to work towards academic goals and created a *“comfortable”* feeling about seeking for help. They believed that they could “*learn better if the atmosphere in the group or in the class is positive and people feel connected to each other*”.

Teachers felt that they would like to play an active role in promoting SoC:

*If the teacher doesn’t contribute, SoC is only created by accident or desperation of the students … But I think that the teacher should give the purpose of the community… by achieving the objective, (students) should be awarded… you (as teacher) foster the community creation… you might inspire, you might push, you might pull.*

When discussing potential barriers to building SoC, all three teachers expressed concern about their time constraints. Further, they felt that promoting SoC would necessitate a shift in roles and a different mindset than a traditional teacher. They were uncertain whether all of their colleagues were ready for the change. Teachers also emphasized the significance of teamwork in this situation, believing that individual teacher efforts would have little impact on raising SoC in BL.

## Discussion

### RQ1: What are teachers’ and students’ perceptions of SoC in BL as promoted through social presence activities?

This study identified teachers’ efforts at developing social presence activities to promote SoC in BL: they attempted to make the courses feel more informal and personalized by incorporating video introductions and humor, they tried using multiple communication channels (e.g., Q&A, discussion boards, and email) to motivate students to share ideas, and they encouraged more reticent students to speak up by providing digital tools for anonymous input. Teachers’ perspectives on how to improve group cohesion varied. Two teachers incorporated collaborative assignments into their courses to foster SoC, while one had yet to do so, possibly due to time constraints.

The significance of non-academic and extracurricular activities in developing SoC is a crucial finding of this study. At the course level, we found that students valued teachers’ efforts to create a welcoming classroom environment in BL; they perceived teachers as “real” and “there” due to personal touches added to a course that helped students connect with teachers and the class as a whole, as also suggested by Lowenthal and Dunlap (2018). Additionally, students required time, space, and opportunities to get to know one another and develop personal rapport. This echoes earlier findings that initial course activities (e.g., icebreakers) can foster the rapid development of trust and assist students in feeling more at ease (Fiock, 2020). Outside class, student desired an additional teacher-free zone (e.g., a WhatsApp group) where they could converse with peers. They greatly valued the support of their programs of study in connecting them across courses, as affinity develops between individuals with similar academic backgrounds. Furthermore, they also viewed the campus as a place where academic and social life can coexist.

### RQ2: What are teachers’ and students’ perceptions of SoC in BL as promoted through cognitive presence activities?

To cultivate students’ cognitive presence, all three teachers in this study provided students with opportunities for independent discovery and exploration of solutions. Videos, texts, and self-evaluated quizzes were frequently used to improve self-study time. To facilitate students’ progress toward higher-order cognitive levels, all teachers incorporated real-world problems into their lessons. Two teachers designed classroom activities to promote reflection, discussion, collaboration, and construction.

According to the students, self-study opportunities that permitted them to comprehend the theory and construct meaning at their own pace were advantageous. They all agreed that group activities contributed significantly to SoC, as these activities maintained their social ties and encouraged them to study together for academic success. In higher education, SoC is more than just bonding relationships; it should also contribute to *learning* (Rovai, 2002b). In line with the existing literature, our study indicates that group purpose and cohesion motivate students to remain dedicated and complete educational tasks collectively (Garrison & Vaughan, 2011). If a course lacks opportunities for group learning, students may form their own groups. For optimal progress toward higher-order learning, we suggest that social and cognitive presence must coexist in the collaborative environment, which conforms with the constructivist view that learning is fundamentally social (Vygotsky & Cole, 1978).

### RQ3: What are teachers’ and students’ perceptions of SoC in BL as promoted through teaching presence activities?

Teachers in this study experimented with various pedagogical and technical methods to enhance SoC. None of the teachers raised concerns regarding technical literacy, possibly because they gained substantial online teaching experience during the pandemic. Post-COVID, teachers continued to explore ways to optimize blended learning, such as improving course design using learning analytics.

Teachers in BL are affected by the educational context in which they work (Jonker et al., 2018). As learning progresses to more challenging cognitive activities, the teacher’s role as a facilitator becomes increasingly crucial for ensuring that students’ contributions are acknowledged and constructive (Garrison & Vaughan, 2011). Our case study results suggest that regardless of class size, subject matter, pedagogical approaches, or prior BL experience, all teachers desire to increase student autonomy and are willing to adapt their roles accordingly. However, despite their desire for relatable teachers, students consider teachers the ultimate authority in the learning process. Some students reported feeling frustrated and uncertain during group activities because they did not receive precise and timely support.

## Recommendations

### Multifaceted support for SoC

According to Cheng (2004), high-quality social life on campus enhances students’ SoC, whereas feelings of isolation negatively affect SoC. We argue that the development of SoC should not be isolated from broader university systems and social-academic contexts. Strategies to enhance SoC can involve expanding the social presence proposed by Garrison et al. (2001) to the program and university levels. To provide comprehensive support for students’ SoC, higher education institutions require a multifaceted infrastructure: teachers concentrate on pedagogical endeavors, while programs and universities can play a significant role in connecting students across different courses. In this situation, the campus's role in fostering an active social and learning environment should be reinforced.

### Digital tools cannot replace in-person contact

This study identified limitations to what the online learning experience can provide, as others have also reported (Reeves et al., 2004).The teachers in this study explored various types of information and communication technology (ICT) in their BL courses. Even though students acknowledge the value of recorded video as a supplement to self-study, they still prefer face-to-face interaction with teachers and peers for complex and in-depth conversations that digital tools cannot adequately replicate. For instance, students perceived discussion boards as a platform for teachers to publish answers, not as a tool to stimulate student engagement, contrary to earlier research's recommendations (Delmas, 2017; Zydney et al., 2012). Additionally, due to “Zoom fatigue” and its potential psychological effects (Bailenson, 2021), we argue that educators must use communication technologies with caution in BL.

### Group work in blended learning is desired but challenging for students

Although our study suggests that students are enthusiastic about group activities in blended learning, they struggle to adapt to group dynamics. They were frustrated and perhaps lacked confidence when working in groups. Interpersonal conflicts, issues with time management, role ambiguity, and cultural differences were identified as obstacles to high-quality group work. As the number of interdisciplinary studies, projects, and international learning opportunities increase in higher education, students may face more significant challenges than ever before when working in groups. Therefore, additional group dynamics training and support for students are recommended.

### Professional development on SoC for teachers is needed

Teachers must strike a delicate balance between allowing students to lead the discussion and providing the direction students request (Garrison & Kanuka, 2004). Because teacher–student rapport is associated with student learning (Wilson et al., 2010), BL must account for the tension caused by students’ and teachers’ divergent viewpoints. In addition, although all three teachers in this study had prior experience developing blended courses, they did not receive training or support for cultivating SoC. Considering teachers’ limited time and team-learning preferences, this void should be filled by future professional development for teachers.

## Conclusion

This study questioned how a sense of community may be established in practice, based on the perspectives of students and teachers in three blended courses. Although we included three cases to increase diversity related to the number of students, teachers’ experience, and the educational fields involved, we acknowledge that this study has limitations because of its small sample size. However, this study did generate several new theoretical propositions that need further investigation.

Our research indicates that all participants recognized SoC's significance for social connection and enhanced learning. First, students highlighted the importance of non-academic activities in establishing SoC that are supported by teachers, programs and university. Second, group learning activities, according to students, significantly sustain SoC by strengthening their mutual motivation to achieve academic success. However, students' difficulties with group dynamics and the divergent viewpoints between students and teachers adversely affected SoC.

This empirical study provides a thick description of social, cognitive, and teaching activities implemented to improve SoC in practice. The holistic analysis of students’ and teachers’ perspectives suggests that the classroom, program, and university as a whole need to be taken into account when seeking to establish SoC. Based on our analysis, we identified a set of recommendations intended to assist practitioners and policymakers in fully leveraging the benefits of blended learning.

## Data Availability

The data used to support the findings of this study are available from the corresponding author upon request.

## Appendix 1. Observation Protocol

This observation protocol is divided into two sections: the first section aims to record the activities classified as social, cognitive, and teaching presence that help students develop a sense of community (SoC). Each presence and its subdivisions are provided with examples.

1. Note down any activities that correspond to the examples provided. Use the *Teacher* and *Student* columns to make additional comments.
2. Use the second section to note down any activities that contributed to SoC, if not possible to be categorized as social, cognitive, or teaching presence.
3. Use the second section to note down any activities that contributed to SoC, but were not facilitated by teachers.

**Table:**

CoI Presence &	Subdivision	Activity *Note down any activity from observation*	Observations	
			Teacher	Student (how the students respond)
Social presence	Affective Expression	Including icebreakers at the beginning of the course		
		Including welcome activities to get to know everyone		
		Using emoticons, humor, and statements in online discussions or other activities		
		Other		
	Open Communication	Utilizing activities that encourage students to share feelings, experiences, interests, and expectations		
		Using a variety of channels to promote communication and interaction		
		Using a discussion board (DB) /forum		
		Other		
	Group Cohesion	Utilizing activities that allow students to work in teams and collaborate on tasks		
		Encouraging students to support each other		
		Other		
Cognitive presence	Triggering Event	Posing questions and problems to increase students’ interest and pique their curiosity		
		Providing tasks to develop students’ problem-solving, reflection, and critical thinking skills		
		others		
	Exploration	Providing a variety of information sources to explore problems		
		Organizing group brainstorming sessions		
		Others		
	Integration	Providing tasks that require combining new information to answer questions or solve problems		
		Providing opportunities for students to reflect on content and discussions		
		Providing students with a variety of activities in which to practice desired skills		
		Others		
	Resolution	Utilizing activities that connect classroom concepts to the real-world examples		
		Providing opportunities for students to apply and practice knowledge to new situations		
		Others		
Teaching Presence	*Instructional design & organization* ** These activities are more likely to be observed during the document analysis phase rather than in a classroom observation*	*Communicating learning goals, topics, expectations, deadlines, *etc*.*		
		Designing content and assignments in diverse of formats (e.g. text, video, quiz, project, readings)		
		Designing and organizing courses to promote interaction and discourse		
		Other		
	Facilitating discourse	Identifying areas of agreement and disagreement on course topics		
		Encouraging and acknowledging student contributions in discussion		
		Providing guidance to help students towards understanding course topics		
		Setting climate for learning		
		Other		
	Direct Instruction (TDI)	Establishing teachers’ personal presence		
		Providing direction instructions by explaining content		
		Helping students focus discussion on relevant issues		
		Confirming students’ understanding for course topics		
		Providing timely and frequent feedback		
		Other		
Other aspects	Activities impacting SoC		Teachers	students

## Appendix 2. Document review checklist

- Scan through the course delivered thought LMS, note down “Yes” if course content, assignment and assessment corresponds to the examples of social, cognitive, and teaching presence and make additional notes if necessary.
- In the “Other aspects” section, note down any course elements that contributed to the SoC, if not possible to be categorized as social, cognitive, or teaching presence.
- In the “Other aspects” section, note down any course elements that contributed to the SoC, but are not facilitated by teachers.

**Table:**

CoI Presence	Subdivision	Examples *Note down any examples of teacher-facilitated activities*	Observations			
			Course Content	Assignment	Assessment	Notes
Social presence	Affective Expression	Including icebreakers at the beginning of the course				
		Including welcome activities to get to know everyone				
		Using emoticons, humor, and statements in online discussions or other activities				
		Other				
	Open Communication	Utilizing course element that encourage students to share feelings, experiences, interests, and expectations				
		Using a variety of channels to promote communication and interaction				
		Using a discussion board /forum				
		Other				
	Group Cohesion	Utilizing course elements allow students to work in teams and collaborate on tasks				
		Encouraging students to support each other				
		Other				
Cognitive presence	Triggering Event	Posing questions and problems to increase students’ interest and pique their curiosity				
		Providing tasks to develop students’ problem-solving, reflection, and critical thinking skills				
		others				
	Exploration	Providing a variety of information sources to explore problems				
		Organizing group brainstorming sessions				
		Others				
	Integration	Providing tasks that require combining new information to answer questions or solve problems				
		Providing opportunities for students to reflect on content and discussions				
		Providing students with a variety of activities in which to practice desired skills				
		Others				
	Resolution	Utilizing activities that connect classroom concepts to the real-world examples				
		Provide opportunities to apply and practice knowledge to new situations				
		Others				
Teaching Presence	*Instructional design & organization*	*Communicating learning goals, topics, expectations, deadlines, *etc*.*				
		Designing content and assignments in diverse of formats (e.g. text, video, quiz, project, readings)				
		Designing and organizing courses to promote interaction and discourse				
		Other				
	Facilitating Discourse	Identifying areas of agreement and disagreement on course topics				
		Encouraging and acknowledging student contributions in discussion				
		Providing guidance to help students towards understanding course topics				
		Setting climate for learning				
		Other				
	Direct Instruction	Establishing teachers’ personal presence				
		Providing direction instructions by explaining content				
		Helping students focus discussion on relevant issues				
		Confirming students’ understanding for course topics				
		Providing timely and frequent feedback				
		Other				
Other aspects	Course elements contributing to SoC					Notes

## Appendix 3

### Interview Protocol for Teachers

*[Read to interviewee]* This interview is part of the research on students’ and teachers' perceptions of a sense of community in a blended learning. The interview will last approximately 60 min. We will start the interview and recording if you do not object.

- Q1. What do you like best about blended learning? Are there any good examples?
- What do you dislike? Do you have any examples?
- Q2. Please describe what ‘2.3’ means to you?
- *[Read to interviewee] According to the literature, sense of community is as a feeling that members have of belonging, a feeling that members matter to one another and to the group, and a shared faith that members' needs will be met through their commitment to be together’*
- Do you have any questions or comments on this definition?
- Q3. How important is it for students to develop a sense of community in blended? Why?
- How important is it for you to contribute to sense of community as a teacher? Why?
- Q4. Do you feel there was a sense of community in the blended courses that you taught? Why? *Alternative: Please give an example of sense of community in the course you taught’ or perhaps where it was missing?*
- Q5. Based on the literature, students can develop a strong sense of community, if teachers organize activities to help them socially connect and collaboratively work together towards learning goals. We noticed that you have tried to use XXX, *[List a few examples of social, cognitive and teaching presence identified from observation and document review. For each example, ask:]*
- How does this help students be more connected with other students?
- How does this help students to learn better?
- Q6.*List a few examples that didn’t occur during the observations and document review.*
- What would convince you to use these as well?
- Q7. What pedagogical methods, practices and tools, in your opinion, have most successfully promoted a sense of community among students? How did it help?
- Are there any other ideas that you would think of to develop a sense of community but haven’t used them yet? What would you need to apply these as well?
- Q8. In terms of supporting SoC, which activities are better suited for f2f? Why?
- Q9. Which activities do you think are best supported online or outside of the classroom?
- Q10. How do you use online technologies to develop a sense of community in blended learning? Are there any examples of improved connectedness and learning outside of the classroom?
- Q11. Optional: What barriers and challenges have you experienced in developing a sense of community in blended learning? *Summarize here what they already said and ask whether there’s anything additional.*
- Q12. Do you receive any training, support, or other opportunities for professional development to promote a sense of community for students?
- Q13. What would help to make you participating in professional development programmes to promote a sense of community in blended learning? What are your wishes? What makes it difficult for you to participate?

!!! Before moving on to the final questions, take a moment to double-check that everything has been covered.

- Q14. Is there anything else we have not mentioned about the sense of community in blended learning that you would like to add?
- Q15. Can I still contact you via email if I have any further questions?

### Interview Protocol for Students

*[Read to interviewee]* This interview is part of the research on students’ and teachers' perceptions of a sense of community in a blended learning environment. The interview will last approximately 60 min. We will start the interview and recording if you do not object.

- Q1. What do you like best about blended learning? Are there any good examples?
- What do you dislike? Any examples?
- Q2. Please describe what ‘2.3’ means to you?
- *[Read to interviewee] According to the literature, sense of community is as a feeling that members have of belonging, a feeling that members matter to one another and to the group, and a shared faith that members' needs will be met through their commitment to be together’*
- Do you have any questions or comments on this definition?
- Q3. As a student, how important is developing a sense of community among students in blended learning to you? Why?
- How important is it for your teacher to contribute to sense of community as a teacher? Why?
- Q4. Do you feel there was a sense of community in the blended courses that you took? Why?
- Q5. Based on the literature, students can develop a strong sense of community if teachers organize activities to help them socially connect and work together towards learning goals. We noticed that your teachers have tried to use XXX, *[List a few examples of social, cognitive and teaching presence identified from observation and document review. For each example, ask:]*
- How does this help you be more connected with other students?
- How does this help you to learn better?
- Q6. *List a few examples that didn’t occur during the observations and document review.*
- Do you think these activities would help you as students to develop a strong sense of community? Do you think there will be any difficulties implementing it?
- Q7. Would you mind sharing anything from this course, in your opinion, has most successfully promoted a sense of community? How did it help?
- What activities do you think can promote a sense of community, but are not used by your teachers or the university? What would you need to apply these as well?
- Q8. In terms of supporting SoC, which activities are better suited for f2f? Why?
- Q9. Which activities do you think are best supported online or outside of the classroom?
- Q10.How do you use online technologies to develop a sense of community in blended learning? Are there any examples of improved connectedness and learning outside of the classroom?
- Q16. What barriers and challenges have you experienced in developing a sense of community in blended learning? *Summarize here what they already said and ask whether there’s anything additional.*
- Q11. Do you think it’s necessary for your teachers to get additional training in promoting a sense of community? Why or why not?

!!! Before moving on to the final question, take a moment to double-check that everything has been covered.

- Q12. Is there anything else we have not mentioned about the sense of community in blended learning that you would like to add?
- Q13. Can I still contact you via email if I have any further questions?

## Appendix 4. Code book

**Table:**

First Level Code	Second Level Code
Social presence	
	Affective Expression
	Open Communication
	Group Cohesion
Cognitive presence	
	Triggering Event
	Exploration
	Integration
	Resolution
Teaching presence	
	Instructional design & organization
	Facilitating discourse
	Direct Instruction
Challenges	
	Group dynamic
	Feelings of frustration
	Motivation
	Limited and late feedback
	Limited guidance and clarity
	Different views between students and teachers
	Hybrid and Covid
	GDPR restrictions
	Technical challenges
	Time constraints
Future work	
	Future work: Professional development input
	Future work: Student wish
	Future work: Teacher wish
Positive experience	
Negative experience	
Teacher's role	
Positive experience	

## Appendix 5. Overview of SoC activities in three courses

✓ available in a course – Not available in the course.

**Table:**

Teacher-led activities	Course A	Student response	Course B	Student response	Course C	Student response
Teaching Presence						
Instructional design & organization						
Flipped classroom	✓	Students appreciated flipped classroom approach for flexible and personalized learning experiences	✓	Students appreciated flipped classroom approach for flexible and personalized learning experiences One student expressed concern as to whether the teacher would review her specific data via learning analytics	✓	Students appreciated flipped classroom for flexible and personalized learning experiences
Learning analytics	–		✓		–	
Facilitating discourse						
Teachers encouraged students to ask questions	✓	Since the course did not explicitly require discussion and collaboration, students wished for more group learning possibilities Students thought DB has the limited value	✓	Students were motivated to come to campus to complete group assignments as well as prepare for exams in groups. Students were actively participating in discussion and making efforts to assist one another	✓	During a F2F class, students discussed, interacted, debated and solved problems together Students thought DB has the limited value
Discussion board (DB)	✓		–		✓	
Incorporate group assignment into F2F sessions	–		✓		✓	
Incorporate group assessment into F2F sessions	–		✓		✓	
Direct Instruction						
Pre-recorded Video	✓	When used for self-study, students found watching movies to be beneficial Students valued the Q&A sessions where the teacher explained subjects and offered help	✓	When used for self-study, students found watching movies to be beneficial Students highly valued supervised tutorials as a way to receive assistance from their teacher But some of them felt like they didn't get enough reassurance from their teacher and wished for more direct answers	✓	When used for self-study, students found watching movies to be beneficial Students liked being able to work on a project with their classmates, but they wished the teacher would be more clear and give them more direction Students perceived project activities in class as "chaotic" at times Some students found peer assessment difficult and they considered that the assessment rubrics should be in charge of the teacher, not the students
Q&A session	✓		–		–	
Supervised tutorial	–		✓		–	
Project meeting & project specialist meeting	–		–		✓	
Social Presence						
Affective Expression						
Greeted students	✓	Students value teachers' efforts to personalize the course. They became more acquainted with their teacher Students in this course came from various academic programmes; they wished to add a social introduction to the beginning of the course	✓	Students from this course came from the same study programme, they felt a natural bond with each other because of their similar academic backgrounds According to the students, a ‘*grea*t’ teacher has a positive impact on their SoC Students appreciated that their programme organized an orientation week for students to become acquainted with one another and their teacher	✓	Students value teachers' efforts to personalize the course. They became more acquainted with their teacher Students in this course came from various academic programmes; they wished to add a social introduction to the beginning of the course. It was difficult for them to form groups on their own and quickly begin working on a subject; they needed more time to "warm up."
Posted welcome messages on LMS	✓		✓		✓	
Personalized course (e.g. intro video)	✓		–		✓	
Teacher joined orientation program	–		✓		–	
Course introduction to get acquainted	–		✓		–	
Open Communication						
Urged students to openly express their ideas	✓	Students considered the limited value of digital communication and preferred in-person contact, this became even more prominent for group discussions or conversations with teachers on difficult and complex topics	✓	Same as course A	✓	Same as course A
Using digital polling	✓		–		–	
Multiple channels for communication (e.g., email, DB and other tools)	✓		✓		✓	
Group Cohesion						
Group assignment	–	Since there were no official group tasks required, students formed an informal group outside of class They expressed struggles with managing group dynamics such as time management conflicts and intercultural communication difficulties	✓	Students felt strong group cohesion, but also expressed struggles with managing group dynamics: not everyone being committed to teamwork and group discussions being dominated by a few group members	✓	Students felt strong group cohesion, but also expressed struggles with managing group dynamics such as role confusion and intercultural communication difficulties
Group assessment	–		✓		✓	
Cognitive Presence						
Triggering Event						
Individual assignments	✓	Students wished for more group assignments Students believed digital polls helped them ‘*reflect on the things*’ they had learned	–	Students enjoyed working together on group tasks. They helped and motivated one another while working on the same tasks	–	Students appreciated the cases based on real-world problems. They enjoyed working together on the final products
Group tasks	–		✓		✓	
Real-world challenges or problem-solving tasks	✓		✓		✓	
Digital poll	✓		–		–	
Exploration						
Online self-study (e.g. watching videos, reading, completing quizzes)	✓	Students highly value the self-study possibilities, they thought their individual learning process was enhanced	✓	Students highly value the self-study possibilities; they thought their individual learning process was enhanced. Students especially like being able to watch videos on Pencast that show how to solve equations	✓	Students said that self-studying was a must if they wanted to understand a subject on their own
Provided ICT tools to support collaboration (e.g. digital conference)	–		–		✓	
Integration & Resolution						
Q&A session	✓	According to students, Q&A sessions helped students the most with their cognitive development. In-depth conversations requiring higher-level thinking from the students often occurred during the Q&A	–	Students took turns explaining the answers and solving the problems together. They helped each other as well as challenged each other They emphasized the significance of coming to campus and meeting face-to-face to engage in in-depth discussions. More complex and difficult questions requiring high-level cognitive development were discussed during F2F sessions	–	Students would need to meet their peers and the teacher to discuss and validate their ideas. These discussion often took place through group assignment and assessment
Group assignment in F2F sessions	–		✓		✓	
Group assessment in F2F sessions	–		✓		✓	
Other than teacher-led activities						
WhatsApp group	✓	All students have their teacher-free zone via a WhatsApp group. They support one another outside of class on academic and non-academic topics They remarked that using WhatsApp on a mobile was considerably more convenient than posting messages on the LMS discussion board	✓	Same as course A	✓	Same as course A
Social activities from study programme	✓	Students were also highly appreciative of the programs' efforts to connect students from different cohorts, allowing them to extend an SoC beyond the confines of a single course	✓	Students appreciated that their programme organized an orientation week for students to become acquainted with one another and their teachers	✓	Students valued the opportunity to join a student association through their programme
Campus social activities	✓	Students acknowledged the importance of campus life because it fostered a sense of belonging and promoted more effective interpersonal communication	✓	Same as course A	✓	Same as course A
